# Charge transfer dependence on CO_2_ hydrogenation activity to methanol in Cu nanoparticles covered with metal–organic framework systems†
†Electronic supplementary information (ESI) available. See DOI: 10.1039/c8sc05441j


**DOI:** 10.1039/c8sc05441j

**Published:** 2019-02-06

**Authors:** Hirokazu Kobayashi, Jared M. Taylor, Yuko Mitsuka, Naoki Ogiwara, Tomokazu Yamamoto, Takaaki Toriyama, Syo Matsumura, Hiroshi Kitagawa

**Affiliations:** a Division of Chemistry , Graduate School of Science , Kyoto University , Kitashirakawa-Oiwakecho, Sakyo-ku , Kyoto , 606-8502 , Japan . Email: hkobayashi@kuchem.kyoto-u.ac.jp ; Email: kitagawa@kuchem.kyoto-u.ac.jp; b JST , PRESTO , 4-1-8 Honcho , Kawaguchi , Saitama 332-0012 , Japan; c Shoei Chemical Inc. , 5-3, Aza-wakazakura Fujinoki-machi , Tosu-shi , Saga 841-0048 , Japan; d Department of Applied Quantum Physics and Nuclear Engineering , Graduate School of Engineering , Kyushu University , Motooka 744, Nishi-ku , Fukuoka , 819-0395 , Japan; e The Ultramicroscopy Research Center , Kyushu University , Motooka 744, Nishi-ku , Fukuoka , 819-0395 , Japan; f Inamori Frontier Research Center , Kyushu University , 744 Motooka, Nishi-ku , Fukuoka , 819-0395 , Japan; g Institute for Integrated Cell-Material Sciences (iCeMS) , Kyoto University , Yoshida, Sakyo-ku , Kyoto , 606-8501 , Japan

## Abstract

We report the synthesis and characterization of highly active Cu nanoparticles covered with zirconium/hafnium-based metal–organic frameworks for CO_2_ hydrogenation to methanol. Compared to **Cu/γ-Al_2_O_3_**, **Cu/ZIF-8**, **Cu/MIL-100** and **Cu/UiO-66** composites, UiO-66 acts as the most active support, with **Cu/Zr-UiO-66** producing methanol at a rate 70 times higher than that of **Cu/γ-Al_2_O_3_**. In addition, the replacement of Zr^4+^ with Hf^4+^ in UiO-66 tripled in the rate of methanol production. Furthermore, we describe a substituent effect on the catalytic activity, with **Cu/Zr-UiO66-COOH** providing a three-fold enhancement of methanol production, compared to that of **Zr-UiO-66** or **Zr-UiO66-NH_2_**. The enhanced catalytic activity of Cu nanoparticles depends on the charge transfer degree from Cu nanoparticles to UiO-66 at the interface between Cu nanoparticles and UiO-66.

## Abstract 2

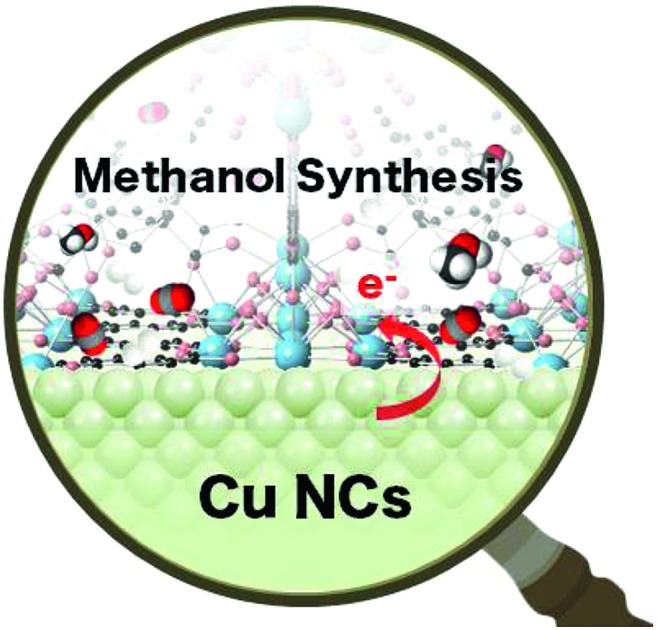
We report the charge transfer dependence on CO_2_ hydrogenation activity to methanol in Cu nanoparticles covered with metal–organic framework systems.

## Introduction

Hybrid materials composed of metal nanoparticles and metal–organic frameworks (MOFs) have attracted much attention for their gas storage, magnetic, optical and catalytic applications due to the remarkable synergistic function between their constituent materials.^1^–^9^ In particular, these hybrid materials have been investigated as highly selective and/or active catalysts because, based on MOF selection, surface properties such as pore size, hydrophobicity/hydrophilicity, and Lewis or Brønsted acidity/basicity can be tuned to control the functionality of the composite.^10^ For example, Liyu Chen *et al.* reported Pd nanoparticles embedded in UiO-67 as a catalyst for selective olefin hydrogenation, with selectivity arising from molecular sieving by the pores of UiO-67.^11^ Kyungsu Na *et al.* reported the hydrogenation of methylcyclopentane to produce selective products depending on the pore size of the Zr-based MOF support.^5^ So far, the strategies on the design for hybrid catalysts of metal nanoparticles and MOFs have been mainly based on the combination of their characteristics. On the other hand, hybrid catalysts with electronic interactions between metal and MOF have received scant attention.^12^–^14^ Since catalytic properties such as activity or selectivity strongly depend on the electronic states of the metal catalysts, tuning the electronic properties of metal nanoparticles by modifying a MOF support is of significance for further development of hybrid catalysts.

Carbon dioxide (CO_2_) conversion into basic chemicals such as formic acid, methanol and carbon monoxide is very important not only for the reduction of CO_2_ emissions, a greenhouse gas and major contributor to global warming, but also for the efficient use of CO_2_ to achieve a carbon neutral energy cycle. In particular, methanol is a good energy carrier as it is a stable liquid under ambient conditions, and it can act as a starting material for the production of olefins, formalin and so on. For these reasons, the development of efficient catalysts for methanol synthesis by CO_2_ hydrogenation has been extensively investigated, yet still remains a challenge as CO_2_ is a stable gas molecule which requires a 6-electron reduction for conversion into methanol. So far, most research on CO_2_ hydrogenation using MOF hybrid materials are related to CO (2-electron reduction) production,^15^,^16^ and there are few reports on CO_2_ hydrogenation to methanol.^7^,^13^ Herein we describe highly active Cu nanoparticle composites for CO_2_ hydrogenation to methanol, with activity controlled *via* charge transfer from Cu to UiO-66 by controlling the functional groups or metal species in the MOF for the first time.

For the MOF component of the composite material, we focused on Zr- and Hf-based UiO-66 (**Zr-UiO-66** and **Hf-UiO-66**, respectively), which consist of hexanuclear M^4+^ oxy/hydroxy clusters linked into a cubic 3-dimensional network by terephthalate linkers. These MOFs were chosen because they have superior thermal, water and chemical stabilities, and because they are easily functionalized through linker substitution or defect formation.^17^–^19^ For functionalization of the terephthalate, we selected carboxylate and amino groups. Carboxylate and amino groups are known to work as electron withdrawing and donating groups to benzene ring, respectively. Therefore, the electronic states of the Zr_6_ cluster in UiO-66 are likely influenced by their substitution groups, which may lead to controlling the degree of charge transfer between Cu nanoparticles and UiO-66.

## Results and discussion

### Synthesis and characterization of **Cu/UiO-66** composite catalysts

We synthesized **Cu/UiO-66** composite catalysts by thermal decomposition of copper acetylacetonate, Cu(acac)_2_ as a Cu precursor in the presence of the pre-synthesized MOF (see experimental details, ESI†). In a typical synthesis, **Zr-UiO-66** was firstly prepared by minor modifications to a solvothermal method reported previously.^19^ The obtained **Zr-UiO-66** and Cu(acac)_2_ were then dispersed in an acetone solution, and the mixture was stirred at room temperature for 24 h. After impregnation, the solvent was removed by centrifugation. The collected solid, which included **Zr-UiO-66** and Cu(acac)_2_, was heated at 350 °C for 1 h under vacuum to obtain the composite of Zr-UiO-66 and Cu nanoparticles (**Cu/Zr-UiO-66**) (see experimental details, ESI†). **Cu/Hf-UiO-66**, **Cu/Zr-UiO-66-NH_2_**, with a 2-aminoterephthalate linker, **Cu/Zr-UiO-66-COOH** with a 1,2,4-benzenetricarboxylate linker, and a γ-Al_2_O_3_ supported Cu catalyst (**Cu/γ-Al_2_O_3_**) as a standard control catalyst were also prepared by the same method to compare their catalytic activity with the non-functionalized **Cu/Zr-UiO-66**.

The amounts of Cu loaded into **Cu/Zr-UiO-66**, **Cu/Zr-UiO-66-NH_2_**, **Cu/Zr-UiO-66-COOH**, **Cu/Hf-UiO-66** and **Cu/γ-Al_2_O_3_** were determined to be 14, 19, 19, 12 and 13 wt%, respectively, by inductively coupled plasma mass spectrometry. TEM images of the composites revealed that Cu nanoparticles are well dispersed and the mean diameters were estimated to be 13.1 ± 3.9, 18.6 ± 5.1, 19.6 ± 4.0, 15 ± 4.1 and 24 ± 4.1 nm for **Cu/Zr-UiO-66**, **Cu/Zr-UiO-66-NH_2_**, **Cu/Zr-UiO-66-COOH**, **Cu/Hf-UiO-66** and **Cu/γ-Al_2_O_3_**, respectively (Fig. S1†).

**Fig. 1:**
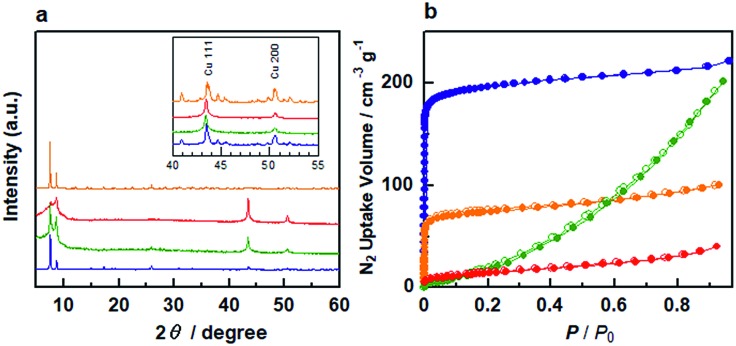
(a) PXRD patterns and (b) N_2_ adsorption/desorption isotherms at 77 K for **Cu/Zr-UiO-66** (blue), **Cu/Zr-UiO-66-NH_2_** (green), **Cu/Zr-UiO-66-COOH** (red) and **Cu/Hf-UiO-66** (orange).

Fig. 1a shows the powder X-ray diffraction (PXRD) patterns of **Cu/Zr-UiO-66**, **Cu/Zr-UiO-66-NH_2_**, **Cu/Zr-UiO-66-COOH** and **Cu/Hf-UiO-66**. The PXRD patterns of all composite materials consist of the diffraction from both Cu and the corresponding UiO-66. **Cu/Zr-UiO-66-NH_2_** and **Cu/Zr-UiO-66-COOH** showed weak diffraction peaks from the MOF component (Fig. S2†), which suggests a decrease in crystallinity, likely arising from some instability of these MOFs at the high temperatures needed to decompose Cu(acac)_2_ (Fig. S3†).

In order to confirm the porosity of UiO-66 after hybridization with Cu nanoparticles, N_2_ sorption isotherms of the composites were measured at 77 K (Fig. 1b). **Cu/Zr-UiO-66** and **Cu/Hf-UiO-66** show typical type-I sorption behavior originating from the microporosity of the MOF, with a decrease in total uptake *versus* the Cu free MOF due to the presence of Cu nanoparticles. In contrast, a sharp decrease in the microporosity is observed in **Cu/Zr-UiO-66-NH_2_** and **Cu/Zr-UiO-66-COOH** corresponding to the lower crystallinity of these samples, which is consistent with the PXRD results. The calculated BET surface areas for **Cu/Zr-UiO-66**, **Cu/Hf-UiO-66**, **Cu/Zr-UiO-66-NH_2_** and **Cu/Zr-UiO-66-COOH** were 702, 207, 172 and 52 m^2^ g^–1^, respectively, in comparison to 1368, 1427, 1074 and 201 m^2^ g^–1^ for the Cu free MOFs (Fig. S4†). From the extended X-ray absorption fine structure analysis, we confirmed that the local structures of Z_6_-clusters in **Cu/UiO-66** composite catalysts were maintained after hybridization with Cu nanoparticles (Fig. S5†).

**Fig. 2:**
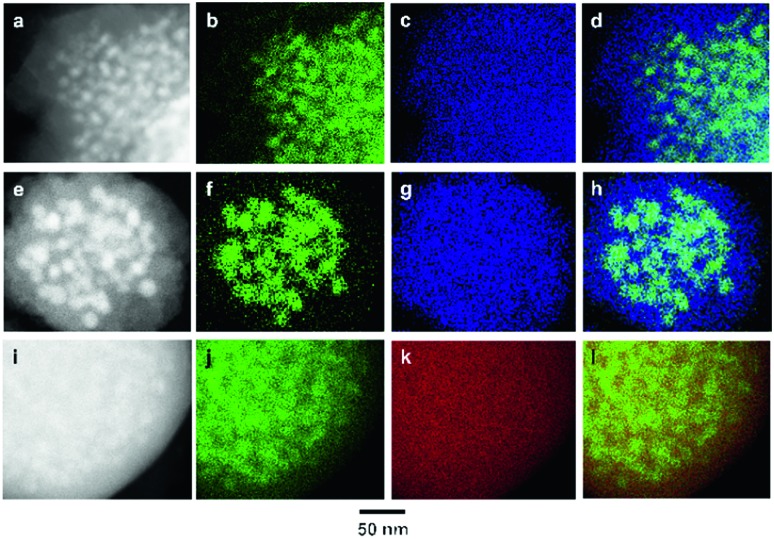
(a) HAADF image, (b) Cu–*K* and (c) Zr–*L* STEM-EDX maps of **Cu/Zr-UiO-66**. (d) Reconstructed overlay image of the maps shown in panels (b) and (c) (green, Cu; blue, Zr). (e) HAADF image, (f) Cu–*K* and (g) Zr–*L* STEM-EDX maps of **Cu/Zr-UiO-66-COOH**. (h) Reconstructed overlay image of the maps shown in panels (f) and (g) (green, Cu; blue, Zr). (i) HAADF image, (j) Cu–*K* and (k) Hf–*L* STEM-EDX maps of **Cu/Hf-UiO-66**. (l) Reconstructed overlay image of the maps shown in panels (j) and (k) (green, Cu; red, Hf).

In order to investigate the composite structure and elemental distribution in **Cu/Zr-UiO-66**, **Cu/Hf-UiO-66** and **Cu/Zr-UiO-66-COOH**, we performed high-angle annular dark-field scanning TEM (HAADF-STEM) and energy-dispersive X-ray (EDX) elemental mapping of Cu and the constituent elements of UiO-66 (Fig. 2). HAADF–STEM images and STEM–EDX mappings of **Cu/Zr-UiO-66**, **Cu/Zr-UiO-66-COOH** and **Cu/Hf-UiO-66** samples are shown in Fig. 2a–d, Fig. 2e–h and Fig. 2i–l, respectively. As shown in Fig. 2a, the HAADF–STEM image of **Cu/Zr-UiO-66** revealed that the MOF covers the Cu nanoparticles. Fig. 2b and c show the corresponding Cu–*K* and Zr–*L* maps which are the constituent metal elements of **Cu/Zr-UiO-66**, and Fig. 2d presents an overlay map of Cu and Zr. These mapping data clearly show that the Zr elements of UiO-66 are distributed around the surface of the Cu nanoparticles. Similarly, HAADF-STEM images and STEM-EDX mappings of **Cu/Zr-UiO-66-COOH** and **Cu/Hf-UiO-66** showed the same results as that of **Cu/Zr-UiO-66**. These results demonstrate that **Cu/Zr-UiO-66** and the analogues are hybrid structures where Cu nanoparticles are covered with UiO-66.

In order to address the possible formation mechanism on Cu nanoparticles covered with UiO-66, we performed the STEM-EDX mapping of the mixture of Zr-UiO-66 and Cu(acac)_2_ after impregnation. The STEM-EDX mapping revealed that the Cu precursor was located inside the pores of Zr-UiO-66 (Fig. S6†). The N_2_ sorption isotherm at 77 K indicated the decrease in the microporosity of Zr-UiO-66 due to incorporation of Cu(acac)_2_ (Fig. S7†). The PXRD patterns of the mixture of Zr-UiO-66 and Cu(acac)_2_ suggested that Cu(acac)_2_ exists in an isolated state (Fig. S8†). Considering that the mean diameter of Cu nanoparticles (13.1 nm) in **Cu/Zr-UiO-66** is larger than that of the pore size of UiO-66 (*ca.* 1 nm), Cu(acac)_2_ loaded into the pores of Zr-UiO-66 is thermally decomposed to generate Cu atoms, and the Cu atoms migrate to form Cu nanoparticles by Ostwald ripening while partially eroding UiO-66. Consequently, Cu nanoparticles are preferentially present in the MOF particles.

### CO_2_ hydrogenation activity to methanol in **Cu/UiO-66** composite catalysts

**Fig. 3:**
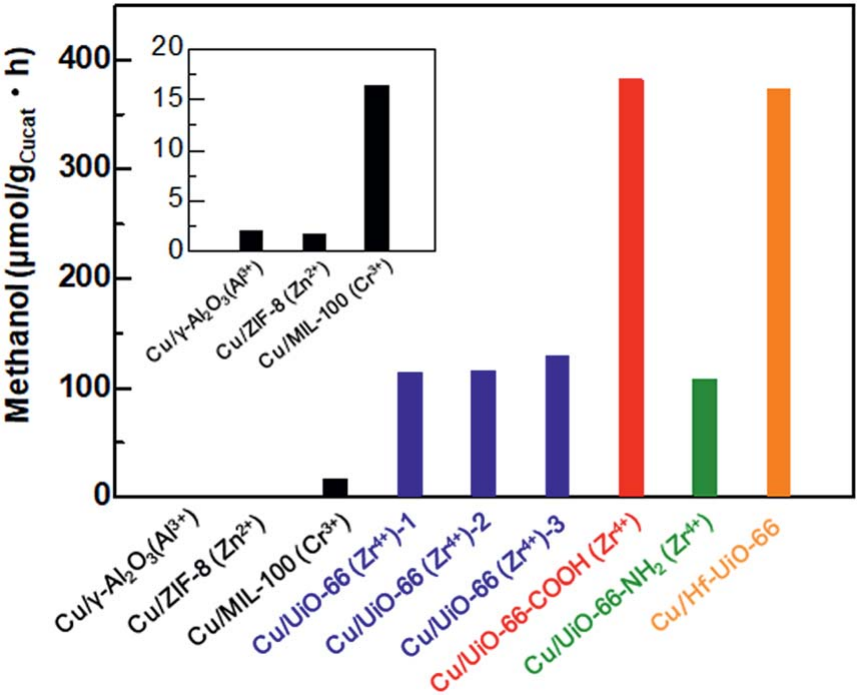
The amount of methanol synthesized from CO_2_ and H_2_ using Cu/γ-Al_2_O_3_ and Cu/MOF composite catalysts. From elemental analysis and TGA, the amount of defects were estimated to be [Zr_6_O_4_(OH)_4.6_(BDC)_5.7_], [Zr_6_O_4_(OH)_4_(BDC)_4.5_(AcO)_3_] and [Zr_6_O_4_(OH)_4_(BDC)_3.6_(AcO)_4.8_] for **Zr-UiO-66-1**, **Zr-UiO-66-2** and **Zr-UiO-66-3**, respectively. AcO = acetate.

To investigate the catalytic activities of the **Cu/UiO-66** composites for CO_2_ hydrogenation to methanol, we performed the activity tests using a fixed bed flow reactor with a gas mixture of 20 sccm CO_2_, 100 sccm H_2_ and 20 sccm He, 2 atm at 220 °C (see experimental details, ESI†). The reaction temperature is lower than the thermally decomposed temperature of UiO-66 and the analogues (Fig. S3†). The amount of methanol synthesized by **Cu/γ-Al_2_O_3_** and Cu/MOFs are shown in Fig. 3. The results of Cu composite catalysts with other well-known MOFs such as ZIF-8 and MIL-100 are also shown (Fig. S9 and 10†). It has been previously reported that Cu shows poor activity for the conversion of CO_2_ into methanol.^20^,^21^ In our experiment, **Cu/γ-Al_2_O** also produced extremely small amounts of methanol (1.9 μmol g_Cu_^–1^ h^–1^) (Fig. 3 inset). On the other hand, **Cu/Zr-UiO-66** exhibited a high catalytic activity, with a rate of methanol synthesis 70 times larger than that of **Cu/γ-Al_2_O_3_** (114.2 μmol g_Cu_^–1^ h^–1^). For comparison, pure Zr-UiO-66 does not show any catalytic activity for CO_2_ hydrogenation. Taking into consideration that **Cu/ZIF-8** and **Cu/MIL-100** have poor CO_2_ hydrogenation activities of 1.8 μmol g_Cu_^–1^ h^–1^ and 16.4 μmol g_Cu_^–1^h^–1^, respectively (Fig. 3 inset), this result indicates that **Zr-UiO-66** demonstrates a highly effective support effect for the Cu catalyst.

It has recently been reported that the defects in MOF play an important role for controlling properties such as gas sorption,^22^ proton conductivity,^23^ optical properties^24^,^25^ and liquid phase catalytic activity.^26^–^28^ Furthermore, UiO-66 is well known to have widely controllable defect concentrations depending on the synthetic conditions.^19^ To investigate a defect effect on the catalytic activity of gas phase CO_2_ hydrogenation, we synthesized **Cu/Zr-UiO-66-1**, **Cu/Zr-UiO-66-2** and **Cu/Zr-UiO-66-3** with increasing amounts of acetic acid to introduce increasing amounts of defects into the Zr_6_-clusters (see experimental details and Fig. S11–S14†). Note: **Cu/Zr-UiO-66** discussed above corresponds to **Cu/Zr-UiO-66-2**. As shown in Fig. 3, all of **Cu/Zr-UiO-66-1**, **Cu/Zr-UiO-66-2** and **Cu/Zr-UiO-66-3** exhibited high catalytic activities, compared to **Cu/γ-Al_2_O_3_**, but provided similar rates of methanol synthesis (114.2, 115.5 and 128. μmolg_Cu_^–1^ h^–1^ for **Cu/Zr-UiO-66-1**, **Cu/Zr-UiO-66-2** and **Cu/Zr-UiO-66-3**, respectively). These results indicate that the reactivity of CO_2_ is not strongly affected by the defects in Zr_6_-clusters of UiO-66. In addition, the N_2_ sorption isotherms of **Cu/Zr-UiO-66-1**, **Cu/Zr-UiO-66-2** and **Cu/Zr-UiO-66-3** (Fig. S14†) indicated that the porosity of UiO-66 also does not directly affect the catalytic activity.

Ligand substitution in **Cu/Zr-UiO-66** seemed to have a major effect on the rate of methanol synthesis with a nearly 3-fold enhancement using carboxylate functionalized **Cu/Zr-UiO-66-COOH** (381.5 μmol g_Cu_^–1^h^–1^*vs.* 114.2 μmol g_Cu_^–1^ h^–1^), but little effect with the amine functionalized **Cu/Zr-UiO-66-NH_2_** (107.7 μmol g_Cu_^–1^ h^–1^). Metal substitution for Hf in **Cu/Hf-UiO-66** also caused a nearly 3-fold enhancement compared to **Cu/Zr-UiO-66** (373.2 μmol g_Cu_^–1^ h^–1^*vs.* 114.2 μmol g_Cu_^–1^ h^–1^). The catalytic activities of **Cu/UiO-66-COOH** and **Cu/Hf-UiO-66** were higher than that of the commercially used Cu/ZnO/γ-Al_2_O_3_ system.^29^ In addition, all of the catalysts (**Cu/Zr-UiO-66**, **Cu/Zr-UiO-66-NH_2_**, **Cu/Zr-UiO-66-COOH** and **Cu/Hf-UiO-66**) gave very high selectivity of over 95% for methanol (Fig. S15†), which is in good agreement with the reported results.^13^

In general, the catalytic activity tends to increase with increasing BET surface area of the catalysts because the reactants can more easily access the surface of catalysts. The BET surface areas of **Cu/Hf-UiO-66** and **Cu/Zr-UiO-66-COOH** in this report were much smaller than that of **Cu/Zr-UiO-66**, although they exhibited significantly enhanced catalytic activities. In addition, the mean diameters of Cu nanoparticles in **Cu/Hf-UiO-66** and **Cu/Zr-UiO-66-COOH** are slightly larger than that of **Cu/Zr-UiO-66**. These results demonstrate that the surface area of UiO-66 does not directly affect the CO_2_ hydrogenation activity, but rather the functional group or metal species is a significant factor. In addition, considering that Cu nanoparticles deposited on UiO-66 produced a smaller amount of methanol (67.8 μmol g_Cu_^–1^ h^–1^) (Fig. S16†), compared with that of **Cu/Zr-UiO-66-2**, the interface between Cu and UiO-66 is also critical to the enhanced catalytic activity.

**Fig. 4:**
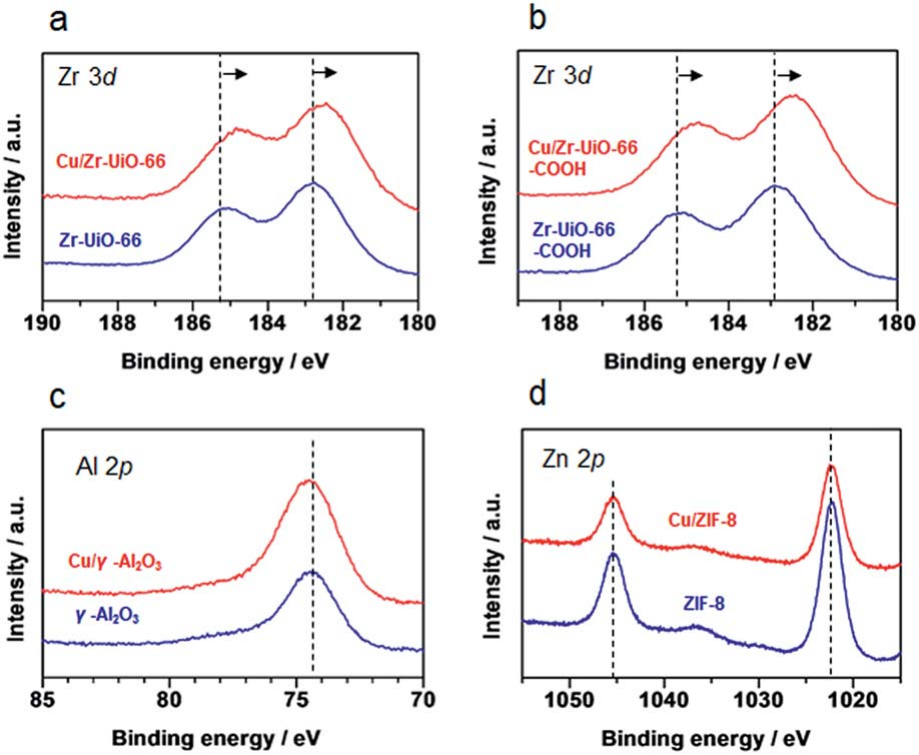
XPS spectra of γ-Al_2_O_3_ and MOF before and after the hybridization with Cu nanoparticles. (a) **Cu/Zr-UiO-66**, (b) **Zr-UiO-66-COOH**, (c) γ-Al_2_O_3_ and (d) **ZIF-8**.

**Fig. 5:**
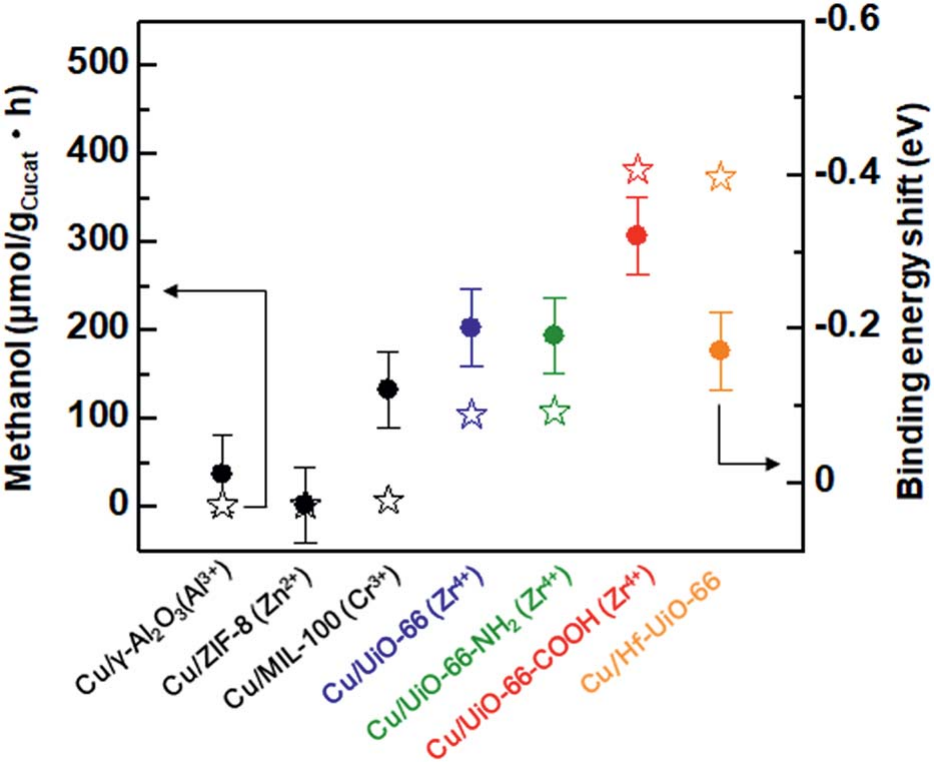
Relationship between the synthesized methanol and binding energy shift estimated by XPS analysis for Cu/γ-Al_2_O_3_ and Cu/MOF catalysts. Star and circle correspond to synthesized methanol and binding energy shift, respectively.

It has been reported that charge transfer between metal nanoparticles and MOF plays an important role in altering the surface/bulk properties of nanoparticles.^1^,^12^–^14^ To investigate possible charge transfer between Cu and MOF, we performed X-ray photoelectron spectroscopy (XPS) of the MOF before and after the hybridization with Cu nanoparticles. As shown in Fig. 4a and b, for **Cu/UiO-66** and **Cu/UiO-66-COOH**, the Zr 3*d* binding energies shifted to lower binding energy after hybridization with Cu nanoparticles, which indicates that Zr^4+^ is in a partially reduced state. In contrast, the Cu 2*p* binding energies of **Cu/UiO-66** and **Cu/UiO-66-COOH** shifted to higher energies with UiO-66 coating, although the shifts became obscured due to the surface oxidation of Cu upon exposure to air (Fig. S17†). These results suggest that charge transfer from Cu nanoparticles to **UiO-66** or **Cu/UiO-66-COOH** occurs in the composites. On the other hand, in the cases of **Cu/γ-Al_2_O_3_** and **Cu/ZIF-8** (Fig. 4c and d), the binding energy shifts of Al 2p and Zn 2p were not observable before and after the hybridization with Cu nanoparticles. The relationship between the produced methanol and the binding energy shift estimated by XPS analysis for **Cu/γ-Al_2_O_3_** and Cu/MOF catalysts is shown in Fig. 5 (Fig. S18 and Table S1†). As shown in Fig. 5, we can clearly see a correlation between the charge transfer and the amount of the produced methanol – the negative shift of the MOF contributes to the catalytic activity for CO_2_ hydrogenation to methanol. In particular, among **Zr-UiO-66** and the analogues such as **Zr-UiO66-NH_2_** and **Zr-UiO66-COOH**, the largest negative shift is observed in **Zr-UiO66-COOH**, which provided the largest rate of methanol synthesis (314.3 μmol g_Cu_^–1^h^–1^). These results are the first demonstration of altering the catalytic activity of Cu nanoparticles *via* charge transfer from Cu to UiO-66 by controlling the functional group or metal species in the MOF. **Hf-UiO-66** showed a smaller binding energy shift (–0.17 eV), compared to **Zr-UiO66-COOH** (–0.32 eV) although the **Cu/Hf-UiO-66** composite demonstrates a catalytic activity comparable with the most active **Cu/UiO-66-COOH**. This is because the binding energy shift of Hf 4f is considered to be relatively smaller than that of Zr 3d due to the valence change.^30^

The PXRD and TEM measurements revealed that the pristine structures of the Cu/UiO-66 catalysts were maintained after the CO_2_ hydrogenation test (Fig. S19 and S20†). Furthermore, after 5 cycles, the catalytic performance of **Cu/Zr-UiO66-COOH** had no obvious change (Fig. S21†), indicating stability of **Cu/Zr-UiO66-COOH** with excellent catalytic performance.

From theoretical calculations,^31^ the rate-determining step for CO_2_ conversion into methanol is hydrogenation of formate, so the stabilization of formate is key to produce methanol at a high rate. It is also known that cationic Cu species helps to stabilize the intermediates (formate).^32^ Therefore, in our system, cationic Cu species due to charge transfer from Cu to the metal components of UiO-66 is considered to promote the stabilization of formate, leading to the enhancement of catalytic activity for CO_2_ conversion into methanol.

## Conclusions

In summary, we first demonstrated the charge transfer dependence on CO_2_ hydrogenation activity to methanol in Cu/MOF systems. Compared to **Cu/γ-Al_2_O_3_**, **Cu/ZIF-8**, **Cu/MIL-100** and **Cu/UiO-66** composites, UiO-66 is demonstrated to have the most highly effective support effect for CO_2_ hydrogenation to methanol, and **Cu/Zr-UiO-66** produced methanol at a rate 70 times larger than that of **Cu/γ-Al_2_O_3_**. The activity of CO_2_ hydrogenation to methanol did not strongly correlate to the amount of defects in the Zr_6_-clusters of UiO-66. On the other hand, the replacement of Zr^4+^ with Hf^4+^ in UiO-66 tripled the rate of methanol production. Furthermore, we found a substituent effect to the catalytic activity, with **Cu/Zr-UiO66-COOH** providing a 3-fold increase in the rate of methanol produced compared to that of **Zr-UiO-66** or **Zr-UiO66-NH_2_**. The charge transfer from Cu to UiO-66 is considered to play an important role in these enhancements of the catalytic activity of Cu. We hope that the results in this study will contribute to the further development of effective catalysts for CO_2_ hydrogenation to methanol, as well as to the future design of highly novel catalysts based on control of the charge transfer between metal nanoparticles and MOF by careful selection of the functional group and/or metal species.

## Conflicts of interest

There are no conflicts to declare.

## Supplementary Material

Supplementary information
