# Supplementary material for: Charge transfer dependence on CO2 hydrogenation activity to methanol in Cu nanoparticles covered with metal–organic framework systems
Source: Chem Sci. 2019 Feb 6;10(11):3289–94. doi: 10.1039/c8sc05441j (PMC6429599; doi:10.1039/c8sc05441j)
Supplement: Supplementary file 1 [file SC-010-C8SC05441J-s001.pdf]

Electronic Supplementary Material (ESI) for Chemical Science.
This journal is © The Royal Society of Chemistry 2019

Electronic supplementary information

Charge Transfer Dependence on CO_2 Hydrogenation Activity into Methanol in Cu Nanoparticles Covered with Metal−Organic Framework Systems

Hirokazu Kobayashi,[a,b] Jared M. Taylor,[a,+] Yuko Mitsuka,[c] Naoki Ogiwara,[a] Tomokazu Yamamoto,[d,e] Takaaki Toriyama,[e] Syo Matsumura,[d,e,f] Hiroshi Kitagawa*[a,f,g]*

a. Division of Chemistry, Graduate School of Science, Kyoto University, Kitashirakawa-Oiwakecho, Sakyo-ku, Kyoto, 606-8502, Japan.

b. JST, PRESTO, 4-1-8 Honcho, Kawaguchi, Saitama, 332-0012, Japan.

c. SHOEI CHEMICAL INC., 5-3, Aza-wakazakura Fujinoki-machi, Tosu-shi, Saga 841-0048 (Japan)

d. Department of Applied Quantum Physics and Nuclear Engineering, Graduate School of Engineering, Kyushu University, Motooka 744, Nishi-ku, Fukuoka, 819-0395, Japan.

e. The Ultramicroscopy Research Center, Kyushu University, Motooka 744, Nishi-ku, Fukuoka, 819-0395, Japan.

f. Inamori Frontier Research Center, Kyushu University, 744 Motooka, Nishi-ku, Fukuoka, 819-0395, Japan.

g. Institute for Integrated Cell-Material Sciences (iCeMS), Kyoto University, Yoshida, Sakyo-ku, Kyoto, 606-8501, Japan.

+ Present address: Department Of Chemistry, University of Calgary, 2500 University Dr. NW, Calgary, Alberta T2N 1N4 (Canada)

E-mail: hkobayashi@kuchem.kyoto-u.ac.jp, kitagawa@kuchem.kyoto-u.ac.jp

Synthesis of UiO-66 and the analogues

Zr-UiO-66: In a typical synthesis, DMF was added to a mixture of the $ZrCl_4$ and acetic acid additive and sonicated to dissolve, forming a slightly cloudy colourless solution. Next, the terephthalic acid was added to the solution and again sonicated to dissolve. Finally, water was added, and the solution was placed into a glass pressure vessel, sealed, and heated in an oven at 120 °C for 24 hours. Following synthesis, the powdery white product was recovered by vacuum filtration and washed with DMF, ethanol, and acetone. To ensure complete exchange of pore solvent/impurities with water, the samples were soaked in acetone (3 x 20 mL) followed by ultra-high-purity water (3 x 20 mL) for one day each in a centrifuge tube and were recovered each time by centrifugation. Finally, the samples were dried in an oven at ~60°C for 24 hours.

**Sample #**	**Terephthalic acid (mg)**	**$ZrCl_4$ (mg)**	**DMF (mL)**	**Acetic acid (mL)**	**Water (μL)**
**Zr-UiO-66-1**	687.3	881.2	67.5	0	200
**Zr-UiO-66-2**	663.8	874.6	63.5	4	200
**Zr-UiO-66-3**	664.0	849.3	51.0	15.0	200

Zr-UiO-66-NH_2: DMF (60 ml) was added to a mixture of the $ZrCl_4$ (866.6 mg) and acetic acid (7.5 ml) additive and sonicated to dissolve, forming a slightly cloudy colourless solution. Next, the 2-aminoterephthalic acid (690.6 mg) was added to the solution and again sonicated to dissolve. Finally, water (200 μL) was added, and the solution was placed into a glass pressure vessel, sealed, and heated in an oven at 120 °C for 24 hours. After the synthesis, the same washing process was performed as the Zr-UiO-66.

Zr-UiO-66-COOH: The carboxylic acid functionalized UiO-66 was synthesized using a reflux method in water modified from a paper.[1] 1,2,4-benzenetricarboxylic acid (3.5601 g) was added to 90 mL of water in a round bottomed flask and stirred. Separately, $ZrCl_4$ (2.3024 g) was dissolved in 10 mL of water. The $ZrCl_4$ solution was then added to the carboxylic acid solution and the solution was set to reflux for 16 hours. After reflux, the solution was cooled, and the product was isolated by centrifugation, washed with deionized water twice, then added to 100 mL of water and set to reflux again for 16 hours. After this the sample was again cooled and isolated by centrifugation, then washed again with water twice.

Hf-UiO-66: In a typical synthesis, DMF (60 ml) was added to a mixture of the $HfCl_4$ (1.1 g) and acetic acid (4.0 ml) additive and sonicated to dissolve, forming a slightly cloudy colourless solution. Next, the terephthalic acid (570 mg) was added to the solution and again sonicated to dissolve. Finally, water (200 μL) was added, the solution was stirred, placed into a glass pressure vessel, sealed, and heated in

a pre-heated oven at 120 °C for 24 hours. After the synthesis, the same washing process was performed as the Zr-UiO-66.

Cu/Zr-UiO-66 and the analogues: The composite materials were synthesized by thermal decomposition of copper acethylacetonate, Cu(acac)$_2$ as a Cu precursors in the presence of the MOF. In a typical synthesis, UiO-66 (1 g) and Cu(acac)$_2$ (824 mg) were dispersed in an acetone solution (100 ml), and the mixture was stirred at RT for 12 h. After the impregnation, the solid was collected by centrifugation. The solid including UiO-66 and Cu(acac)$_2$ was then heated at 350 ^oC for 1h under vacuum to produce the composite of UiO-66 and Cu nanoparticles (Cu/UiO-66). The UiO-66 the analogues and γ-Al_2O_3 supported Cu catalyst (Cu/γ-Al_2O_3) were also prepared by the same method.

Cu nanoparticles deposited on UiO-66 (Cu on UiO-66): For the preparation of **Cu on UiO-66**, Cu(acac)$_2$ (824 mg) and UiO-66(1 g) were gained by agate mortar for 10 min, and the mixture was then heated at 350 ^oC for 1h under vacuum.

Transmission electron microscopy (TEM) images: TEM images were captured using a Hitachi HT7700 instrument operated at 100 kV accelerating voltage.

High-resolution STEM images and STEM-EDX mapping images: High-resolution STEM images and STEM-EELS mapping images were captured using a JEOL JEM-ARM200F STEM instrument operated at 200 kV accelerating voltage.

Powder X-ray diffraction (PXRD) measurements: The structures of MOF and Cu/MOF hybrid catalysts were investigated by powder XRD analysis using a Bruker D8 Advance diffractometer (Cu $K\alpha$ radiation).

Inductively coupled plasma-mass spectrometry (ICP-MS): The weignt % of Cu included in hybrid catalysts was estimated using a Shimadzu ICPE-9000 instrument.

N_2 sorption isotherms: N_2 adsorption/desorption isotherms were measured using a BELSORP-max (Microtrac BEL) at 77 K up to 1 bar. Before starting the adsorption measurements, each sample was activated by heating under vacuum at 120 °C for 12 h.

Catalytic tests and condition: The catalytic tests were performed using a fixed bed flow reactor (Microtrac BEL), which was connected to a gas chromatograph (Shimadzu GC-2014). The amount of Cu/MOF catalyst loaded in the reactor was 0.3 g. The catalytic test was performed using a gas mixture of 20 sccm CO_2, 100 sccm H_2 and He 20 sccm at 220 ^oC, 2 atm. As pretreatment of catalysts, the gas

mixture was flowed for 10 h before the catalytic tests. The produced methanol was analyzed by flame ionization gas chromatography.

Thermogravimetry analysis (TGA): The TGA analysis of samples was performed using a NETZSCH Japan TGA-DTA 2,000SA with a heating rate of 5 K per min under a constant N_2 flow.

X-ray photoelectron spectroscopy (XPS) spectra: The XPS spectra of samples on a carbon sheet were recorded using an ESCA-3400 (Shimadzu). The binding energies were corrected by referencing the C(1s) line at 284.3 eV.

XAS measurements. X-ray absorption spectra (XAS) were collected at the BL14B2 beam line, SPring-8 in transmission mode under ambient conditions, using a Si (311) double crystal monochromator. The data were processed with IFEFFIT. Fourier transformation was k^3-weighted in the k range from 3.0 to 14.0 Å^{-1} for Zr *K*-edge, and from 3.0 to 16.0 Å^{-1} for Hf L_{III}-edge. Before the measurements, the sample treatment was performed at 340 ^{o}C for 3 h under H_2 gas for 10 h.

Defect Estimation. The defect estimation was performed following previously reported method.[2] Thermogravimetric analysis was performed in alumina pans under air from 20 °C to 600 °C at a rate of 5 K per min. In case of **UiO-66-1** synthesized without acetic acid, defect formula was calculated by analysing the mass loss in the decomposition step progressing from a formula of $Zr_6O_{6+x}(C_8H_4O_4)_{6-x}$ at 400 °C to $6ZrO_2$ at 550 °C with an expected UiO-66/$6ZrO_2$ ratio of 2.20 for x = 0. The x value was 0.3 (Figure SX). Elemental analysis was also used to quantify the defects using a formula of $Zr_6O_{6+x}(C_8H_4O_4)_{6-x}\cdot yH_2O$. The values of x and y for the samples were 0.3 and 21 for 1 (calc. 3.49% H, 27.34% C, found 3.37% H, 27.21% C). In cases of **UiO-66-2,3** synthesized with acetic acid, defect formulae were calculated by analysing the mass loss in the decomposition step progressing from a formula of $ZrO(C_8H_4O_4)_{1-x}(C_2H_3O_2)_{2x}$. at 320 °C to $6ZrO_2$ at 550 °C with an expected UiO-66/$6ZrO_2$ ratio of 2.20 for x = 0. The x values were 1.5 and 2.4 for **UiO-66-2** and **UiO-66-3**, respectively. Elemental analysis were also used to quantify the defects using a formula of $Zr_6O_6(C_8H_4O_4)_{6-x}(O_2C_2H_3)_{2x}\cdot yH_2O$. The values of x and y for the samples were 1.5 and 10 for **UiO-66-2** (calc. 2.89% H, 28.41% C, found 2.77% H, 28.39% C), and 2.4 and 5 for **UiO-66-3** (calc. 2.62% H, 28.06% C, found 2.93% H, 28.31% C).

Results and Discussion

1. TEM images

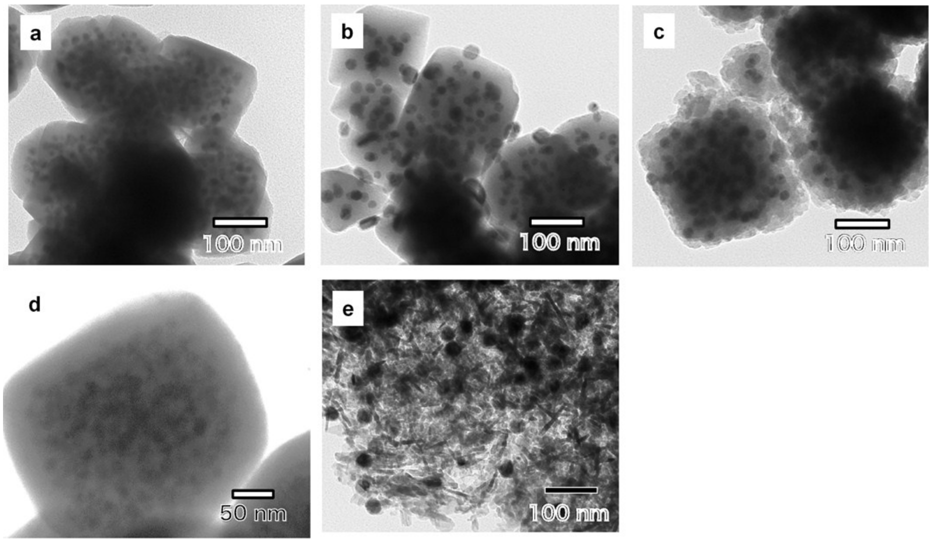

Figure S1 TEM images of (a) **Cu/Zr-UiO-66**, (b) **Cu/Zr-UiO-66-NH$_2$**, (c) **Cu/Zr-UiO-66-COOH**, (d) **Cu/Hf-UiO-66** and (e) **Cu/*γ*-Al$_2$O$_3$.**

2. PXRD patterns

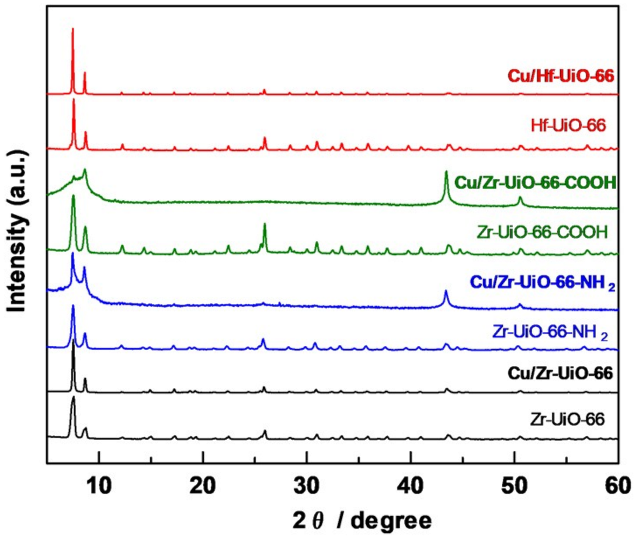

Figure S2. PXRD patterns of UiO-66, **Cu/Zr-UiO-66** and their analogues.

3. TGA

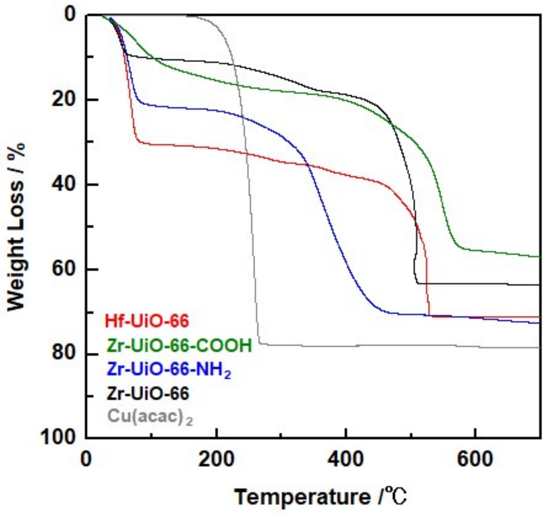

Figure S3. TGA of $Cu(acac)_2$, UiO-66, and the analogues.

4. N_2 sorption isotherms at 77 K for UiO-66 and the analogues

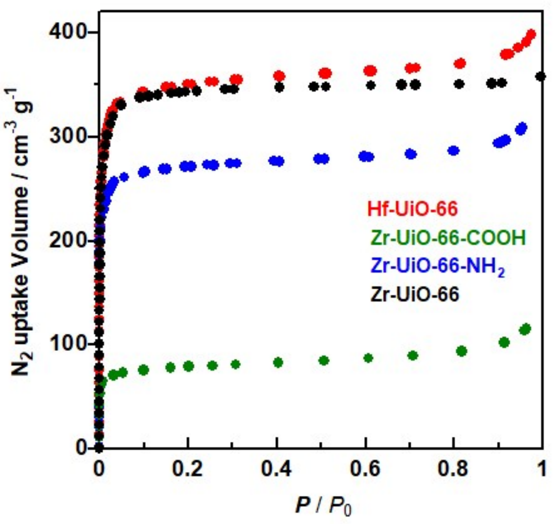

Figure S4. N_2 sorption isotherms of UiO-66 and the analogues.

5. FT-EXAFS spectra of UiO-66 and Cu/UiO-66 samples

In UiO-66, **Cu/Zr-UiO-66-1** and **Cu/Zr-UiO-66-2**, the peaks observed at 1.7 A and 3.2 A are assignable to Zr-O and Zr-Zr in inorganic $Zr_6O_4(OH)_4(CO_2)_{12}$ cluster of UiO-66 (hydroxylated strcuture).[3] In **Cu/Zr-UiO-66-3**, **Cu/Zr-UiO-66-NH$_2$** and **Cu/Zr-UiO-66-COOH**, the peaks observed at 2.9 A are assignable to Zr-Zr in inorganic $Zr_6O_6(CO_2)_{12}$ cluster of UiO-66 (dehydroxylated strcuture),[3] in

addtion to the peak at 1.7 A originating from Zr-O. The FT-EXAFS spectra demonstrated that the local structures of Z_6-clusters are maintained after hybridization with Cu nanoparticles. From the results of EXAFS for **Cu/Zr-UiO-66-1**, **Cu/Zr-UiO-66-2** and **Cu/Zr-UiO-66-3**, it was found that $Zr_6O_4(OH)_4(CO_2)_{12}$ and $Zr_6O_6(CO_2)_{12}$ clusters of UiO-66 do not directly affect the CO_2 hydrogenation activities (Fig. 3).

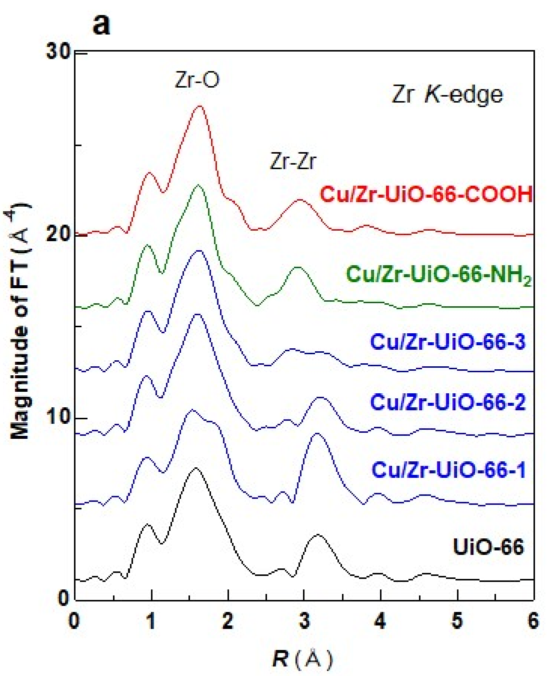

Figure S5. (a) Zr *K*-edge and (a) Hf L_{III}-edge EXAFS spectra in *R* space showing the magnitude of Fourier Transform for UiO-66 and Cu/UiO-66 samples.

6. HAADF image and STEM-EDX maps of the mixture of Zr-UiO-66 and Cu(acac)$_2$ after the impregnation.

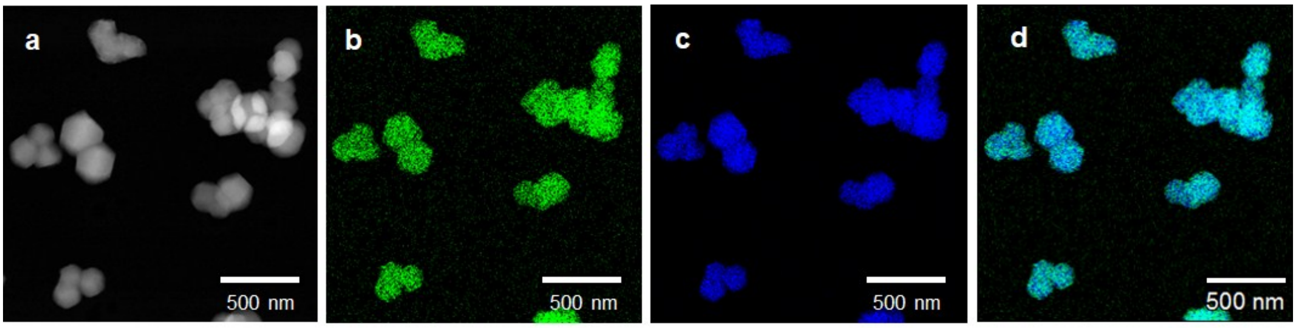

Figure S6. (a) HAADF image, (b) Cu-K, (c) Zr-L STEM-EDX and (d) the overlay maps of (b) and (c) for the mixture of Zr-UiO-66 and Cu(acac)$_2$ after the impregnation.

7. N_2 sorption isotherms at 77 K for the mixture of Zr-UiO-66 and $Cu(acac)_2$ after the impregnation

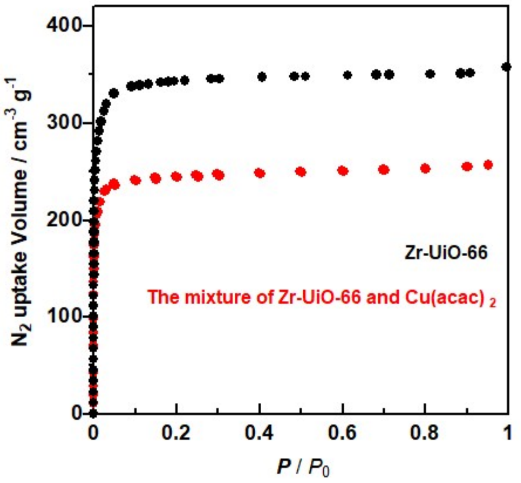

Figure S7. N_2 sorption isotherms for Zr-UiO-66 (black) and the mixture of Zr-UiO-66 and $Cu(acac)_2$ after the impregnation (red).

8. PXRD patterns

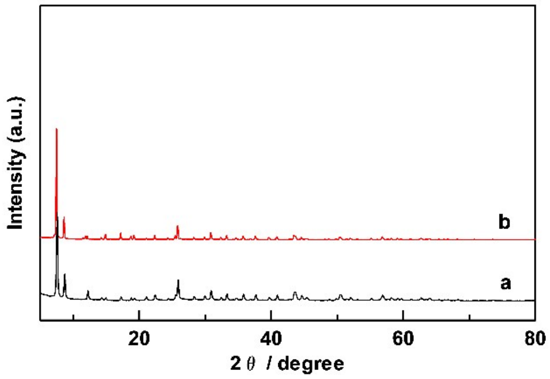

Figure S8. PXRD patterns of (a) Zr-UiO-66 and (b) the mixture of Zr-UiO-66 and $Cu(acac)_2$ after the impregnation.

The synthesis and characterization of Cu/ZIF-8 and Cu/MIL-100

ZIF-8 was provided by BASF as Basolite Z-1200. MIL-100-Cr was prepared by a hydrothermal synthesis. The composite materials were synthesized by thermal decomposition of $Cu(acac)_2$ in the presence of the MOF as is the case with Cu/Zr-UiO-66 and the analogues.

9. TEM images of Cu/ZIF-8 and Cu/MIL-100

From TEM images of Cu/ZIF-8 and Cu/MIL-100, the diameter of Cu nanoparticles were estimated to be 38 nm and 45 nm for **Cu/ZIF-8** and **Cu/MIL-100**, respectively.

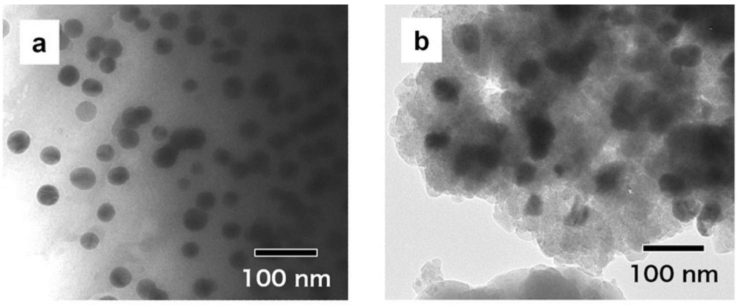

Figure S9. TEM images of **Cu/ZIF-8** and **Cu/MIL-100**

10. PXRD patterns of Cu/ZIF-8 and Cu/MIL-100

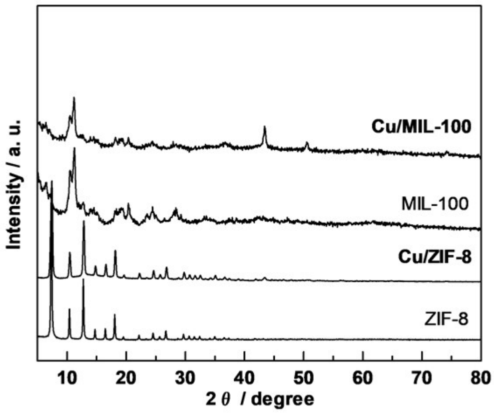

Figure S10. PXRD patterns of **Cu/ZIF-8** and **Cu/MIL-100**

11. TGA of UiO-66-1, UiO-66-2 and UiO-66-3 under air to estimate the defects

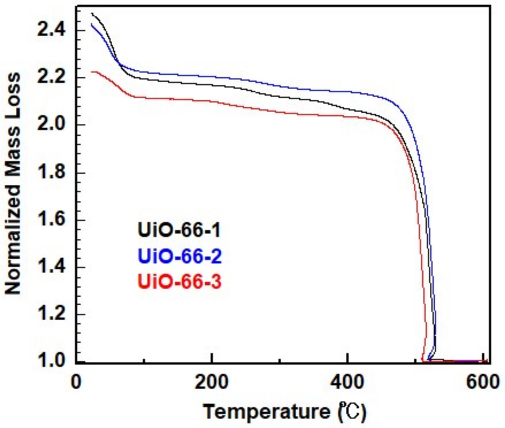

Figure S11. Normalized TGA thermal curves for UiO-66-1, UiO-66-2 and UiO-66-3 under air flow, normalized to the mass of ZrO_2 decomposition product. A normalized mass loss of 2.2 corresponds to a defect free sample (x=0)

12. TEM images of Cu/Zr-UiO-66-1, Cu/Zr-UiO-66-2 and Cu/Zr-UiO-66-3

From TEM images, the mean diameters of Cu nanoparticles in **Cu/Zr-UiO-66-1, Cu/Zr-UiO-66-2** and **Cu/Zr-UiO-66-3** were estimated to be 14.1 ± 3.0 nm, 13.1 ± 3.9 nm and 12.5 ± 2.9 nm, respectively. The amounts of Cu loaded into **Cu/Zr-UiO-66-1, Cu/Zr-UiO-66-2** and **Cu/Zr-UiO-66-3** were determined to be 12, 14 and 12 wt%, respectively, by ICP-MS.

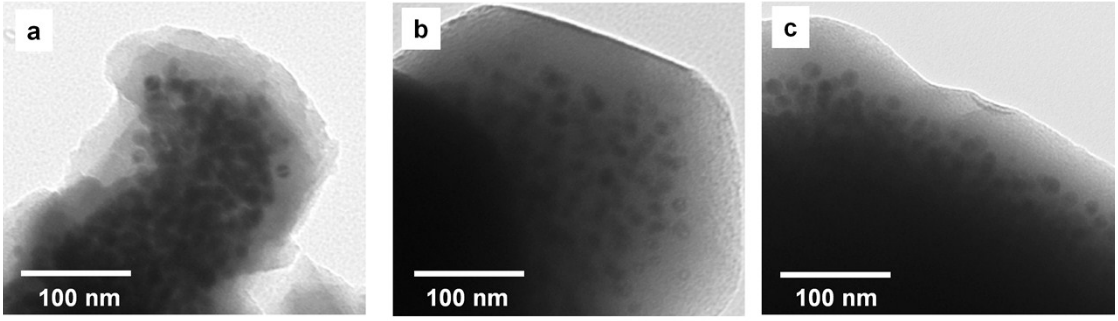

Figure S12. TEM images of (a) **Cu/Zr-UiO-66-1**, (b) **Cu/Zr-UiO-66-2** and (c) **Cu/Zr-UiO-66-3**

13. PXRD patterns of Cu/Zr-UiO-66-1, Cu/Zr-UiO-66-2 and Cu/Zr-UiO-66-3

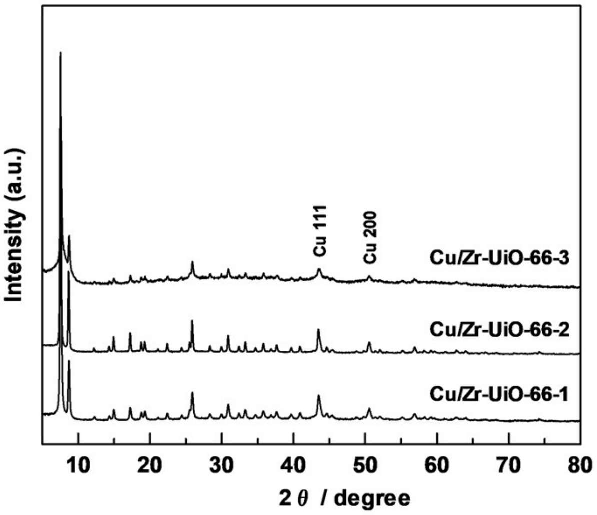

Figure S13. PXRD patterns of **Cu/Zr-UiO-66-1, Cu/Zr-UiO-66-2** and **Cu/Zr-UiO-66-3**

14. N_2 sorption isotherms at 77 K for Cu/Zr-UiO-66-1, Cu/Zr-UiO-66-2 and Cu/Zr-UiO-66-3

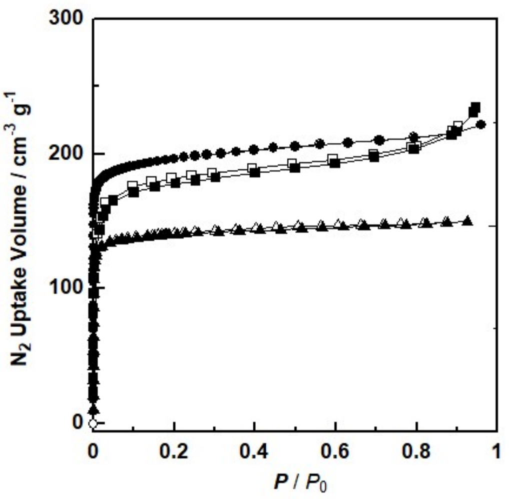

Figure S14. N_2 sorption isotherms of **Cu/Zr-UiO-66-1** (▲; adsorption, △; desorption)**, Cu/Zr-UiO-66-2** (●; adsorption, ○; desorption) and **Cu/Zr-UiO-66-3** (■; adsorption, □; desorption)

15. Product selectivity obtained by Cu/Zr-UiO-66, Cu/Zr-UiO-66-NH_2, Cu/Zr-UiO-66-COOH and Cu/Hf-UiO-66

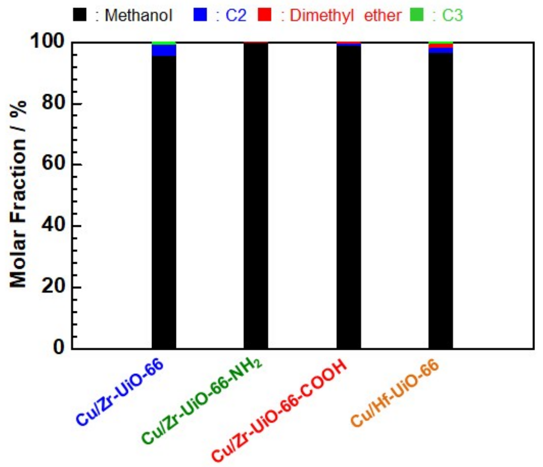

Figure S15. Product selectivity obtained by **Cu/Zr-UiO-66**, **Cu/Zr-UiO-66-NH_2**, **Cu/Zr-UiO-66-COOH** and **Cu/Hf-UiO-66** under the hydrogenation reaction condition.

16. Characterization of Cu on UiO-66

TEM image of the composites revealed that Cu nanoparticles are located on the surface of UiO-66 (Fig. S17a). The mean diameter of Cu nanoparticles was estimated to be 38.0 ± 11.5 nm. The amounts of Cu included in the composite was determined to be 15.6 wt%. The PXRD pattern of the composite composed of the diffraction from both Cu and the corresponding UiO-66 (Fig. S17b). The **Cu on UiO-66** showed typical type-I sorption behavior originating from the microporosity of the MOF, with a decrease in total uptake versus Cu free UiO-66 due to the presence of Cu nanoparticles (Fig. S17c).

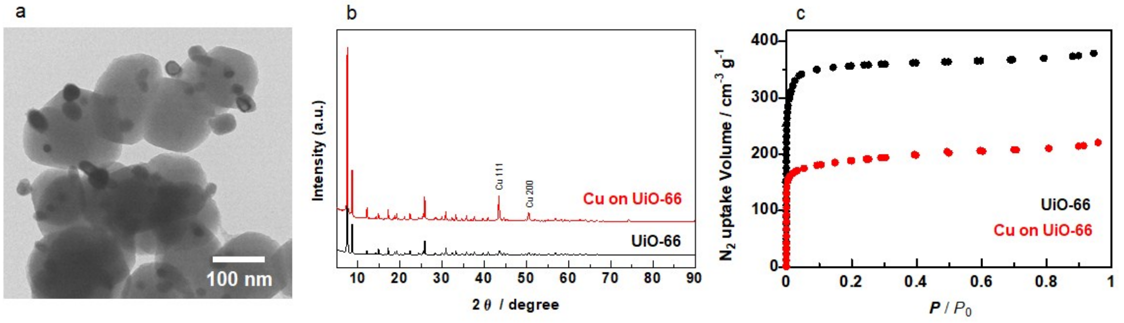

Figure S16. (a) TEM image of **Cu on UiO-66** and PXRD patterns and N_2 sorption isotherms at 77 K of UiO-66 and **Cu on UiO-66**.

17. XPS spectra of Cu nanoparticles in the hybrid catalysts

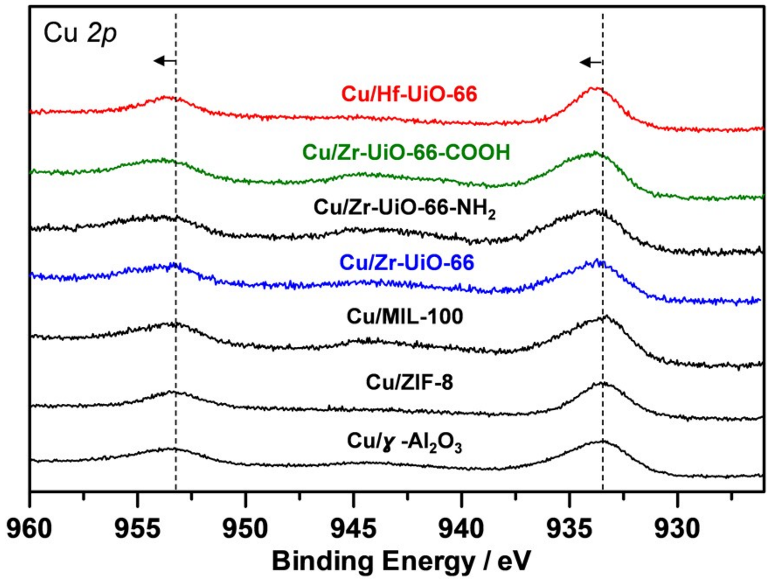

Figure S17. XPS spectra of Cu nanoparticles in the hybrid catalysts

18. XPS spectra of MOF before and after the hybridization with Cu nanoparticles

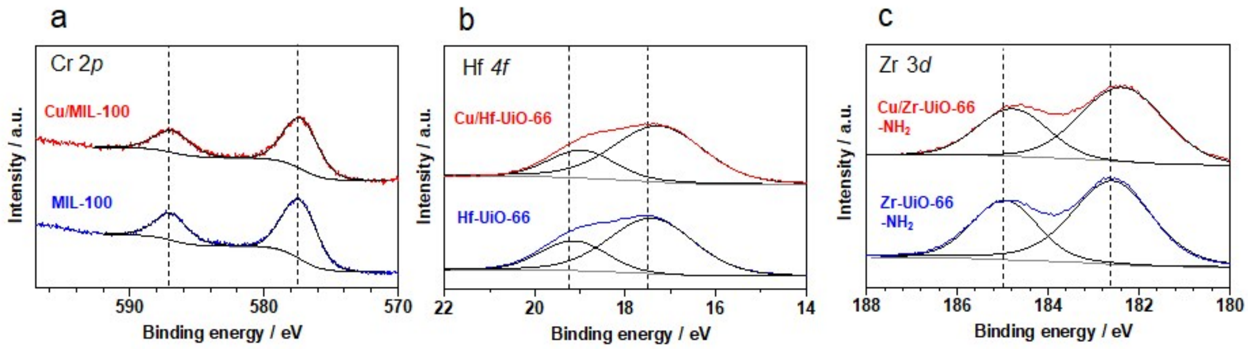

Figure S18. XPS spectra of MOF before and after the hybridization with Cu nanoparticles. (a) **Cu/MIL-100**, (b) **Cu/Hf-UiO-66**, (c) **Cu/UiO-66-NH$_2$**.

Table S1. The synthesized methanol and binding energy shift estimated by XPS analysis for Cu/γ-Al_2O_3 and Cu/MOF catalysts.

Support material	Methanol ($\mu mol/g_{Cucat}\cdot h$)	Binding energy shift (eV) [a]
**γ-Al_2O_3**	**1.5**	**-0.01**
**ZIF-8**	**1.8**	**+0.03**
**MIL-100**	**7.2**	**-0.12**
**Zr-UiO-66**	**104.7**	**-0.20**
**Zr-UiO-66-NH$_2$**	**107.7**	**-0.19**
**Zr-UiO-66-COOH**	**381.5**	**-0.32**
**Hf-UiO-66**	**373.2**	**-0.17**

[a] The difference of binding energy before and after hybridization with Cu nanoparticles.

19. TEM images

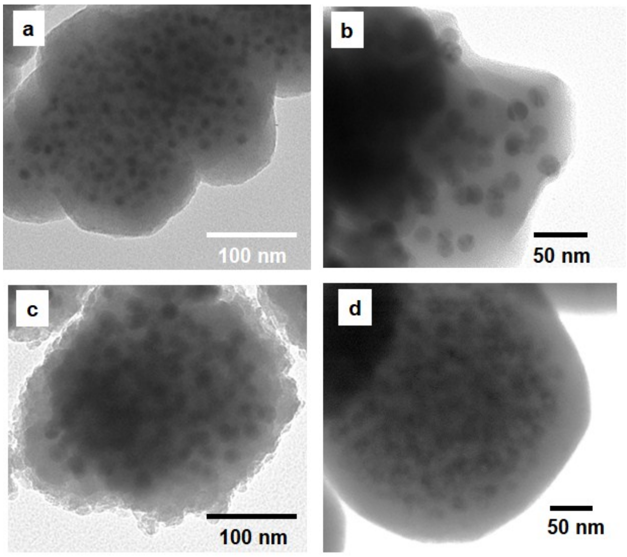

Figure S19 TEM images of (a) **Cu/Zr-UiO-66**, (b) **Cu/Zr-UiO-66-NH$_2$**, (c) **Cu/Zr-UiO-66-COOH,** (d) **Cu/Hf-UiO-66** after the catalytic test**.**

20. PXRD patterns of Cu/Zr-UiO-66 and their analogues before and after the catalytic test.

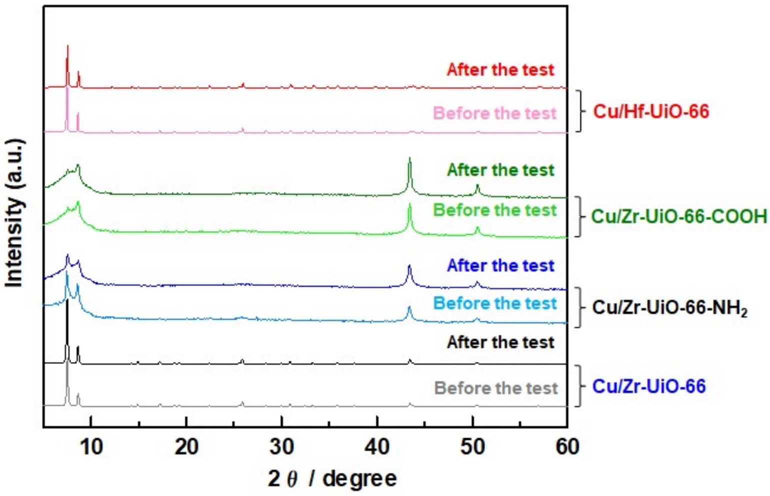

Figure S20. PXRD patterns of **Cu/Zr-UiO-66** and their analogues before and after the catalytic test.

21. The recyclability of Cu/Zr-UiO-66-COOH.

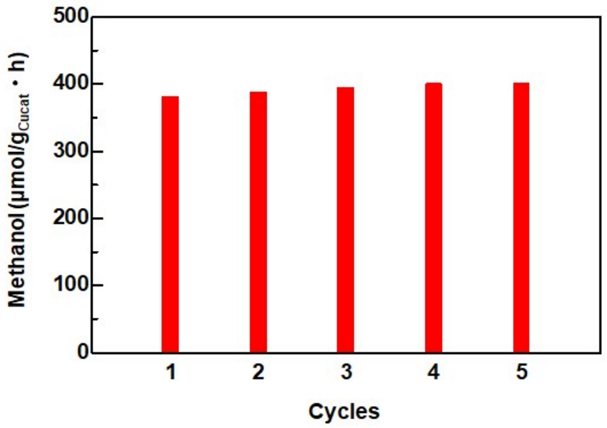

Figure S21. The recyclability of **Cu/Zr-UiO-66-COOH**.

References

[1] Q. Yang, S. Vaesen, F. Ragon, A. D. Wiersum, D. Wu, A. Lago, T. Devic, C. Martineau, F. Taulelle, P. L. Llewellyn, H. Jobic, C. Zhong, C. Serre, G. De Weireld and G. Maurin, *Angew. Chem., Int. Ed.,* 2013*,* **52***,* 10316.

[2] J. Taylor, S. Dekura, R. Ikeda and H. Kitagawa, *Chem. Mater*., 2015, **27**, 2286.

[3] L. Valenzano, B. Civalleri, S. Chavan, S. Bordiga, M. H. Nilsen, S. Jakobsen, K. P. Lillerud and C. Lamberti, *Chem. Mater*., 2011, **23**, 1700.
